# Differential subcellular localization of ASPM RNA and protein

**DOI:** 10.17912/micropub.biology.001080

**Published:** 2024-01-24

**Authors:** Hei-Yong G Lo, Chad G Pearson, J Matthew Taliaferro

**Affiliations:** 1 Department of Biochemistry and Molecular Genetics, University of Colorado Anschutz Medical Campus, Aurora, Colorado, United States; 2 RNA Bioscience Initiative, University of Colorado Anschutz Medical Campus, Aurora, Colorado, United States; 3 Department of Cell and Developmental Biology, University of Colorado Anschutz Medical Campus, Aurora, Colorado, United States; 4 Linda Crnic Institute for Down Syndrome, University of Colorado Anschutz Medical Campus, Aurora, Colorado, United States

## Abstract

RNAs encoding some centrosomal components are trafficked to the organelle during mitosis. Some RNAs, including *ASPM* , localize to the centrosome co-translationally. However, the relative position of these RNAs and their protein after trafficking to centrosomes remained unclear. We find that mislocalization of *ASPM* RNA from the centrosome does not affect the localization of ASPM protein. Further, *ASPM* RNA and ASPM protein reside in two physically close yet distinct subcellular spaces, with *ASPM* RNA on the astral side of the centrosome and ASPM protein on the spindle side. This suggests subtly distinct locations of *ASPM* RNA translation and ASPM protein function.

**Figure 1. ASPM RNA and protein localization:**
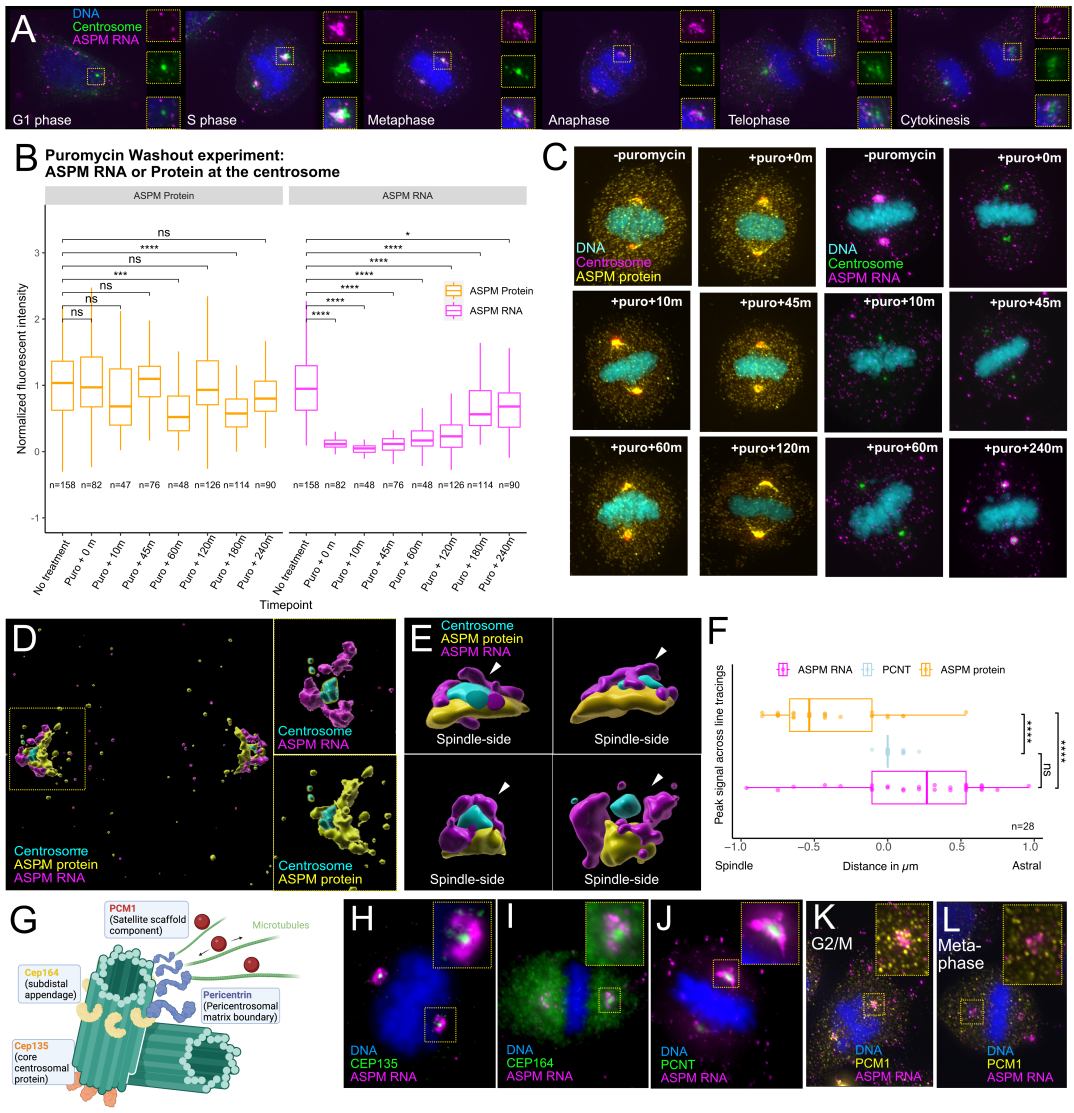
**(A)** Fluorescence microscopy of HeLa cells stained with smFISH for *ASPM* RNA (magenta) in relation to the centrosome (Halo-Tagged PCNT; green) across the cell cycle (60x). Insets show individual signals and merge. Fluorescence intensity was treated equally for all cell cycle stages. **(B)** Quantification of *ASPM* RNA (purple) and ASPM protein (yellow) around the centrosome after puromycin treatment and washout. Times reflect the amount of time after removal of puromycin. N represents a single centrosome where RNA and protein were quantified. **(C)** Representative fluorescence images of metaphase cells in puromycin washout experiment. Relative *ASPM* RNA (magenta) or ASPM protein (yellow) surrounding the centrosome (Halo-Tagged PCNT; green) is visualized (60x). Times reflect the amount of time after puromycin treatment. Fluorescence intensity was treated equally for all cell cycle stages. **(D)** Representative metaphase cells were imaged (100x, SIM) and reconstructed with Imaris software. Reconstructions show the differential localization of *ASPM* RNA (magenta), ASPM protein (yellow), and the centrosome (cyan) in relation to one another. Insets show either RNA or protein in relation to a centrosome. **(E)** Four additional centrosomes rendered as in D, with astral *ASPM* RNA (magenta) highlighted with white arrows. **(F)** Bar graphs depicting the peak signal intensities for each line tracing of either ASPM protein, ASPM RNA or PCNT. Each data point represents the peak signal of a single line tracing of a single centrosome. N represents a single centrosome **(G)** Diagram depicting the relative location of different centrosome proteins. **(H-J)** Fluorescence images of *ASPM* RNA (magenta) in relation to centrosome markers (green) CEP135 (H), CEP164 (I), and PCNT (J) in metaphase cells (60x). **(K, L)** Fluorescence images of cells in G2/M (K) or metaphase (L) depicting the relative localization of *ASPM* RNA in relation to PCM1 (yellow) (60x). Two-sided student T-tests were used in statistical analyses (n.s. represents p > 0.05, * represents p < 0.05, ** represents p < 0.01, *** represents p < 0.001, **** p < 0.0001).

## Description

The centrosome is a dynamic structure. Depending on the cell type and cell stage, the organelle serves numerous functions, from ciliogenesis to mitosis (Woodruff, Wueseke, and Hyman 2014; Nigg and Raff 2009) . This flexibility is facilitated by changing the content of proximal proteins and RNAs (Woodruff et al. 2017; Sonnen et al. 2012) . In recent years, various groups have identified nine RNAs that localize to the human centrosome during mitosis (Chouaib et al. 2020; Sepulveda et al. 2018; Safieddine et al. 2021; Forrest and Gavis 2003; Bergalet et al. 2020) . One such RNA encodes abnormal spindle-like microcephaly-associated protein or ASPM. *ASPM* RNA localizes to the centrosome only during mitosis ( **Figure 1A** ). Like other centrosome-proximal RNAs, the localization of *ASPM* RNA to the centrosome during mitosis has been shown to be co-translational (Chouaib et al. 2020; Safieddine et al. 2021; Sepulveda et al. 2018) . Since the addition of puromycin, but not cycloheximide, interferes with *ASPM* RNA localization to the centrosome, it has been hypothesized that *ASPM* RNA trafficking requires interaction between the RNA, a translating ribosome, the nascent peptide, and transport machinery (Safieddine et al. 2021; Sepulveda et al. 2018) . However, the subcellular localization of *ASPM* RNA in relation to its encoded protein and other centrosome markers has not been explored.

We first used single molecule RNA fluorescence in situ hybridization (smFISH) to validate previous findings that the localization of *ASPM* RNA to the centrosome of HeLa cells is disrupted following puromycin treatment (10 minutes at 100 µg/mL) ( **Figure 1B, C** ) (Safieddine et al. 2021; Tsanov et al. 2016) . We then asked how long it would take for *ASPM* RNA to relocalize to the centrosome after puromycin washout. Assuming a standard translation speed of 6 amino acids per second (Prabhakar et al. 2017; Dana and Tuller 2012) , the complete translation of the ASPM open reading frame should take approximately 10 minutes. We therefore expected the relocalization of the RNA to occur shortly following treatment in mitotic cells. Surprisingly, we found that *ASPM* RNA did not relocalize to the centrosome of mitotic cells until 3 hours after puromycin washout and did not recover to baseline levels of centrosome localization even 4 hours after puromycin treatment ( **Figure 1B, C** ). The reason for this delayed relocalization is uncertain. Previous research demonstrated that RNA transport speed is not likely a limiting factor as ASPM RNA polysomes were shown to travel along microtubules at approximately 1 µm/s (Safieddine et al. 2021) . Importantly, the cells were not stabilized in metaphase during the puromycin washout. Rather, the cells in metaphase at these later timepoints (3-4 hours) reflect cells that were previously in G2/S phase when treated with puromycin. This result raises the possibility of a cell cycle-phase specific window where the transport machinery for *ASPM* RNA is assembled approximately 3 hours prior to metaphase.

Since *ASPM* RNA at the centrosome has been demonstrated to be locally translated (Safieddine et al. 2021) , we next asked whether mislocalization of *ASPM* RNA affected localization of its encoded protein. Surprisingly, mislocalization of *ASPM* RNA largely did not result in the mislocalization of ASPM protein ( **Figure 1B, C** ). These results suggest that though co-translation is required to localize *ASPM* RNA, the localization of *ASPM* RNA to the centrosome is not critical for maintaining a centrosome-proximal population of ASPM protein.

During analysis of ASPM protein and RNA at the centrosome, we consistently noted that the ASPM protein and RNA exhibit unique localization patterns. We noted that *ASPM* RNA formed a “ring” around the centrosome (relative to Halo-tagged pericentrin (PCNT)) ( **Figure 1C** ). ASPM protein appeared to localize along the spindle-side of the centrosome ( **Figure 1C** ). To better resolve the ASPM RNA and protein localization, we imaged the two using super resolution, structured illumination microscopy (SIM), which has a resolution limit of approximately 100 nm (Kner et al. 2009) . We found that *ASPM* RNA and its cognate protein consistently localized to proximal yet separate subcellular spaces ( **Figure 1D -E** ). In relation to the center of the centrosome (marked by PCNT), we found that ASPM protein was significantly more localized to the spindle-side of the centrosome. ASPM RNA was present on both sides but biased toward the astral side ( **Figure 1F** ). The population of *ASPM* RNA localized to the astral-side of centrosomes was spatially distinct from its encoded protein ( **Figure 1D -F** ).These results are consistent with our previous finding that while *ASPM* RNA localization is dependent on translation, precise ASPM protein localization may rely on a separate mechanism.

Finally, given the centrosomal localization of *ASPM* RNA, we asked whether we could define the specific subcellular localization of *ASPM* RNA in relation to well-defined markers that label different sub-regions of the centrosome. We visualized *ASPM* RNA in relation to a core component of centrioles (CEP135), a subdistal appendage marking the distal side of centrioles (CEP164), and a component of the pericentriolar matrix (PCNT) ( **Figure 1G** ). *ASPM* RNA localized peripherally to all these centrosome markers ( **Figure 1G -J** ). This placed *ASPM* RNA outside of the pericentriolar matrix. Pericentriolar Material 1 (PCM1) is a scaffold component of the centriolar satellites that transport cargo along microtubules to and from the pericentriolar space (Hames et al. 2005; Hall et al. 2023) . We therefore asked whether *ASPM* RNA resided in the same space as PCM1. We found that *ASPM* RNA resided in the same space as PCM1 in prometaphase ( **Figure 1K** ). Surprisingly, in metaphase, we consistently found that PCM1 is notably absent from centrosomes where *ASPM* RNA still resides ( **Figure 1L** ). This suggests that though PCM1 and *ASPM* RNA reside in a similar pericentrosomal space, *ASPM* RNA localization around the centrosome during metaphase is not dependent on PCM1.

Taken together, these findings affirm that *ASPM* RNA localization to the centrosome occurs co-translationally (Sepulveda et al. 2018; Safieddine et al. 2021) . We further found that following mis-localization, *ASPM* RNA takes three hours to return to the centrosome. The reason for this delay is uncertain. We also found that ASPM protein is not perturbed following *ASPM* RNA mis-localization and ASPM protein localizes to unique pericentrosomal domains compared to *ASPM* RNA. ASPM protein localization to the spindle-side is consistent with the hypothesized role of ASPM protein as a partner for microtubule disassembly (Jiang et al. 2017) . The *ASPM* RNA located on the aster side of the centrosome may indicate that the aster side is more accessible for active trafficking of RNA and other molecules than the spindle side. Alternatively, and not mutually exclusively, it may also indicate other roles for *ASPM* RNA at this location, including potential structural roles for macromolecular organization.

## Methods

*Creation of transgenic cell lines expressing Halo fusion proteins*

Two HeLa cell lines expressing centrosome-localized Halo-tagged (Los et al. 2008) transgenes, Halo-PCNT and Halo-CEP135, were made to visualize the centrosome. These HeLa cells contained a single loxP-flanked expression cassette. Each cell line was therefore created by integrating a plasmid containing the Halo fusion using site specific cre/lox recombination. These cells were plated in a six-well plate to 70% confluence and co-transfected with 2000 ng of plasmid containing the N-terminally tagged HaloTag fusion protein and an antibiotic selectable marker along with 100 ng of a plasmid encoding *cre* recombinase. Cells were transfected with Lipofectamine 2000 (Thermo Scientific) following manufacturer’s instructions. Twenty-four hours after transfection, cells were incubated with blasticidin (5 µg/mL) to select for integrants. Blasticidin selection was used in order to allow cells to be treated and affected by puromycin in translation-inhibition experiments. Cells were maintained on blasticidin, but blasticidin was removed for experiments.

The integrated plasmid also contained a reverse tetracycline-controlled transactivator (rtTA), which placed expression of the Halo fusion under doxycycline control. To induce expression of the Halo fusion, cells were incubated with 1 µg/mL doxycycline for 48 hours prior to performing experiments. Janelia Fluors were used to visualize the Halo fusion (1:2000, Tocris).

*smiFISH oligo pool creation and probe hybridization*

The R script Oligostan (Tsanov et al. 2016) was used to design primary smFISH probes for *ASPM* . Each probe was 26-32nt in length with 40-60% GC content. To increase specificity, 48 designed probes were chosen for *ASPM* . Primary probes were designed with overhanging sequences (TTACACTCGGACCTCGTCGACATGCATT) for hybridization to a secondary oligo containing two Cy3 conjugations. Primary probes were diluted to 20 µM total probe concentration. To hybridize the fluorescent secondary oligonucleotide to the primary oligo pool, the secondary probe was added in 20% molar excess to the primary probes and incubated in NEB buffer 3 in a thermocycler at 85°C for 3 minutes, then cooled to 65°C for 3 minutes, then held at 25°C. For hybridization of these probes to endogenous RNA, 2 µL of these hybridized probes was combined with 100 µL of smFISH hybridization buffer.

*Visualization of both RNA and protein with smFISH and antibody staining*

Standard IF techniques are incompatible with smFISH detection and vice-versa. Therefore, we developed a method to visualize both protein and RNA localization within a cell utilizing a similar technique previously described (Kochan, Wawro, and Kasza 2015) . Plated cells were fixed with 10% formalin. MeOH fixation is not compatible with this protocol, as it limits the antibodies that can be used. Additionally, 70% EtOH is not used in this protocol, as it disrupts primary antibody binding. Cells were permeabilized with PBST at 0.05% for 1 hour at 4°C. Following permeabilization, cells were washed with PBS (not PBST), as Tween interferes with smFISH probe binding to RNA. Cells were then stained with primary antibodies overnight at 4°C. The following antibodies and concentrations were used: (Rabbit anti-ASPM C-terminus (1:500), Novus Biologicals cat no. NB100-2278; Rabbit anti-Cep164 (1:2000), Proteintech cat no. 22227-1-AP; Rabbit anti-PCM1 (1:1000), Thermo Fisher cat no. PA5-54779). The following day, the primary antibody was washed off with PBS. The corresponding secondary antibody was added at RT for 1 hour (Goat Anti-rabbit IgG Alexa Fluor 555, Cell Signaling, cat. no. 4413S; Goat anti-Rabbit IgG Alexa Fluor 647 cat no. A-21245). Cells were then fixed again with 10% formalin for an additional 5 minutes to bind antigens, primary antibodies, and secondary antibodies together. Cells were then incubated with smiFISH wash buffer A (Stellaris) supplemented with 10% formamide. Coverslips were incubated cell-side down in the hybridization buffer, with at least 40 nM of total probe in 50-100 µL of hybridization buffer in a humid homemade hybridization chamber. Coverslips were incubated overnight at 37°C in the dark. Cells were washed the next day with wash buffer A (Stellaris) before being incubated with 100 ng/mL of DAPI in wash buffer A in the dark at 37°C for 15 minutes. Cells were then washed once more with wash buffer B (Stellaris) then mounted onto slides with Fluoromount G and sealed with nail polish. The entire protocol takes 3 days to complete.

*Translation inhibition treatment*

To inhibit translation, HeLa cells were treated with puromycin (100 µg/mL) for 10 minutes. Puromycin was subsequently washed away with PBS and replaced with complete media. Cells were fixed at various time points following removal of translation inhibitors: 0 minutes, 10 minutes, 45 minutes, 1 hour, 2 hours, 3 hours, and 4 hours. All further processing steps for microscopy were identical for each sample.

*Microscopy / Imaging*

Translation-inhibition images were imaged on a widefield DeltaVision Microscope at 60 X (GE) with consistent laser intensity and exposure times across samples. Images were deconvolved using the default Deltavision software SoftWorx. Max projection was created with FIJI with maximum intensity projected. SIM images were collected on a Nikon SIM (N-SIM) with a Nikon Ti2 (Nikon Instruments; LU-N3-SIM) microscope equipped with a 100x SR Apo TIRF, NA 1.49 objective. Images were captured using a Hamamatsu ORCA-Flash 4.0 Digital CMOS camera (C13440) with 0.25µm Z step sizes. Raw SIM images were reconstructed with an image slice reconstruction algorithm. 3D reconstruction was rendered with Imaris software.

*Image analysis*

All images were processed with FIJI (Schindelin et al. 2012) . Representative images are depicted for each sample. All images shown comparing fluorescence intensity were treated identically from acquisition to image processing. The images were taken on the same day with the same exposure times and laser power. Any changes in brightness or contrast were kept consistent across images. Image stacks were maximum intensity projected.

Cells were classified to their cell cycle stage by careful examination of DNA organization, phenotypic features, and fluorescence intensity of DAPI.

Fluorescence intensity of ASPM protein or RNA staining near the centrosome was measured using a circular ROI measuring 2 µm around the centrosome (visualized by Halo-PCNT). RNA and protein staining was quantified at each centrosome measured. A similar ROI was measured within the cell that excluded the centrosomes to calculate in-cell background. Total intensity was calculated by subtraction of background signal with the signal measured at the centrosome. Sample size was based on estimations by power analysis with a significance level of 0.05.

To quantify the localization of ASPM RNA and protein in relation to the centrosome, we performed line tracings on centrosomes stained for all three. A line ROI was drawn from the spindle side to the astral side, extending 1 µm in either direction from the peak PCNT signal (the center of the centrosome). The fluorescence of *ASPM* RNA, ASPM protein, and PCNT was measured across this ROI. The peak fluorescent intensity of each signal for each centrosome was plotted. A two-sided T-test was used to determine differences in the position of peaks across conditions.
